# Middle molecule clearance with high cut-off dialyzer versus high-flux dialyzer using continuous veno-venous hemodialysis with regional citrate anticoagulation: A prospective randomized controlled trial

**DOI:** 10.1371/journal.pone.0215823

**Published:** 2019-04-26

**Authors:** Lorenz Weidhase, Elena Haussig, Stephan Haussig, Thorsten Kaiser, Jonathan de Fallois, Sirak Petros

**Affiliations:** 1 Medical Intensive Care Unit, University Hospital Leipzig, Leipzig, Saxony, Germany; 2 Department of Internal Medicine/Cardiology, University of Dresden, Heart Center Dresden, Dresden, Saxony, Germany; 3 Institute for Laboratory Medicine, Clinical Chemistry and Molecular Diagnostics, University Hospital Leipzig, Leipzig, Saxony, Germany; Osake University Graduate School Of Medicine, JAPAN

## Abstract

**Background:**

Regional anticoagulation with citrate during renal replacement therapy (RRT) reduces the risk of bleeding, extends dialyzer lifespan and is cost-effective. Therefore, current guidelines recommend its use if patients are not anticoagulated for another reason and if there are no contraindications against citrate. RRT with regional citrate anticoagulation has been established in critically ill patients as continuous veno-venous hemodialysis (CVVHD) to reduce citrate load. However, CVVHD is inferior regarding middle molecule clearance compared to continuous veno-venous hemofiltration (CVVH). The use of a high cut-off dialyzer in CVVHD may thus present an option for middle molecule clearance similar to CVVH. This may allow combining the advantages of both techniques.

**Methods:**

In this prospective, randomized, single-blinded single-center-trial, sixty patients with acute renal failure and established indication for renal replacement therapy were randomized 1:1 into two groups. The control group was put on CVVHD using regional citrate anticoagulation and a high-flux dialyzer, while the intervention group was on CVVHD using regional citrate anticoagulation and a high-cut-off dialyzer. The concentrations of urea, creatinine, β_2_-microglobulin, myoglobin, interleukin 6 and albumin were measured pre- and post-dialyzer 1, 6, 12, 24 and 48 hours after initiating CVVHD.

**Results:**

Mean plasma clearance for β_2_-microglobulin was 19.6±5.8 ml/min in the intervention group vs. 12.2±3.6 ml/min in the control group (p<0.001). For myoglobin (8.0±4.5 ml/min vs. 0.2±3.6 ml/min, p<0.001) and IL-6 (1.5±4.3 vs. -2.5±3.5 ml/min, p = 0.002) a higher mean plasma clearance using high-cut-off dialyzer could be detected too, but no difference for urea, creatinine and albumin could be observed concerning this parameter between the two groups.

**Conclusion:**

CVVHD using a high cut-off dialyzer results in more effective middle molecule clearance than that with high-flux dialyzer.

**Trial registration:**

German Clinical Trials Register (DRKS00005254, registered 26th November 2013)

## Introduction

Acute kidney injury requiring renal replacement therapy (RRT) is associated with a high mortality [1, 2] and represents an independent risk factor besides the severity of the underlying disease [3]. Continuous renal replacement therapy (CRRT) offers better hemodynamic stability and gentle removal of solutes and fluids [4]. Nevertheless, a better survival could not be demonstrated with CRRT compared to intermittent hemodialysis (IHD) [5, 6]. Furthermore, there is a lack of evidence concerning optimal dose [7, 8, 4, 9, 10] and the best time point to start RRT [11, 12].

Anticoagulation during CRRT is necessary to avoid blood clotting in the extracorporeal circuit. Systemic anticoagulation poses a risk, particularly in patients with a high risk of bleeding, such as the critical care patient population. Regional citrate anticoagulation has already been implemented in clinical practice during the last years and it has been proved feasible and safe [13]. It reduces the risk of bleeding [14, 15], extends dialyzer lifespan [15, 16] and it is cost-effective [16]. However, a definite survival advantage has not yet been demonstrated [17, 18]. Current international guidelines recommend regional citrate anticoagulation if patients are not anticoagulated for another reason and if there are no contraindications against citrate [19]. Citrate has to be metabolized in the intermediary metabolism to bicarbonate. Thus, its metabolism may be seriously impaired in patients with severe liver dysfunction [20].

A lower extracorporeal circuit blood flow is required in diffusion-based RRT techniques compared to convection-based ones. Therefore, continuous veno-venous hemodialysis (CVVHD) enables reducing citrate load in critical care patients [21]. However, CVVHD is inferior regarding middle molecule clearance compared to continuous veno-venous hemofiltration (CVVH) [22]. Using high cut off (HCO) membranes with a pore size larger than 0.01 μm in CVVHD could be a solution for this problem [23, 24]. One of such dialyzers is the Ultraflux EMiC2 (Fresenius Medical Care, Bad Homburg, Germany). Clinical data concerning this dialyzer are limited.

The aim of this prospective randomized trial was to evaluate the middle molecule clearance with citrate anticoagulated CVVHD using a HCO-dialyzer compared to CVVHD with the standard high-flux dialyzer.

## Material and methods

This study is a prospective, randomized, single-blinded single-center trial in a 28-bed medical intensive care unit (ICU) at the University Hospital Leipzig, Germany. The study was approved by the local ethics committee (University of Leipzig, reference number: 447-12-24092012), conducted in accordance with the German medical product law and registered in the German Clinical Trials Register (DRKS00005254, registered 26 November 2013). Informed consent was obtained from all participating subjects. Eligible patients were enrolled after informed consent by the patients themselves or their legal guardians. The trial was conducted between May 2014 and May 2015.

### Subjects

**Fig 1:**
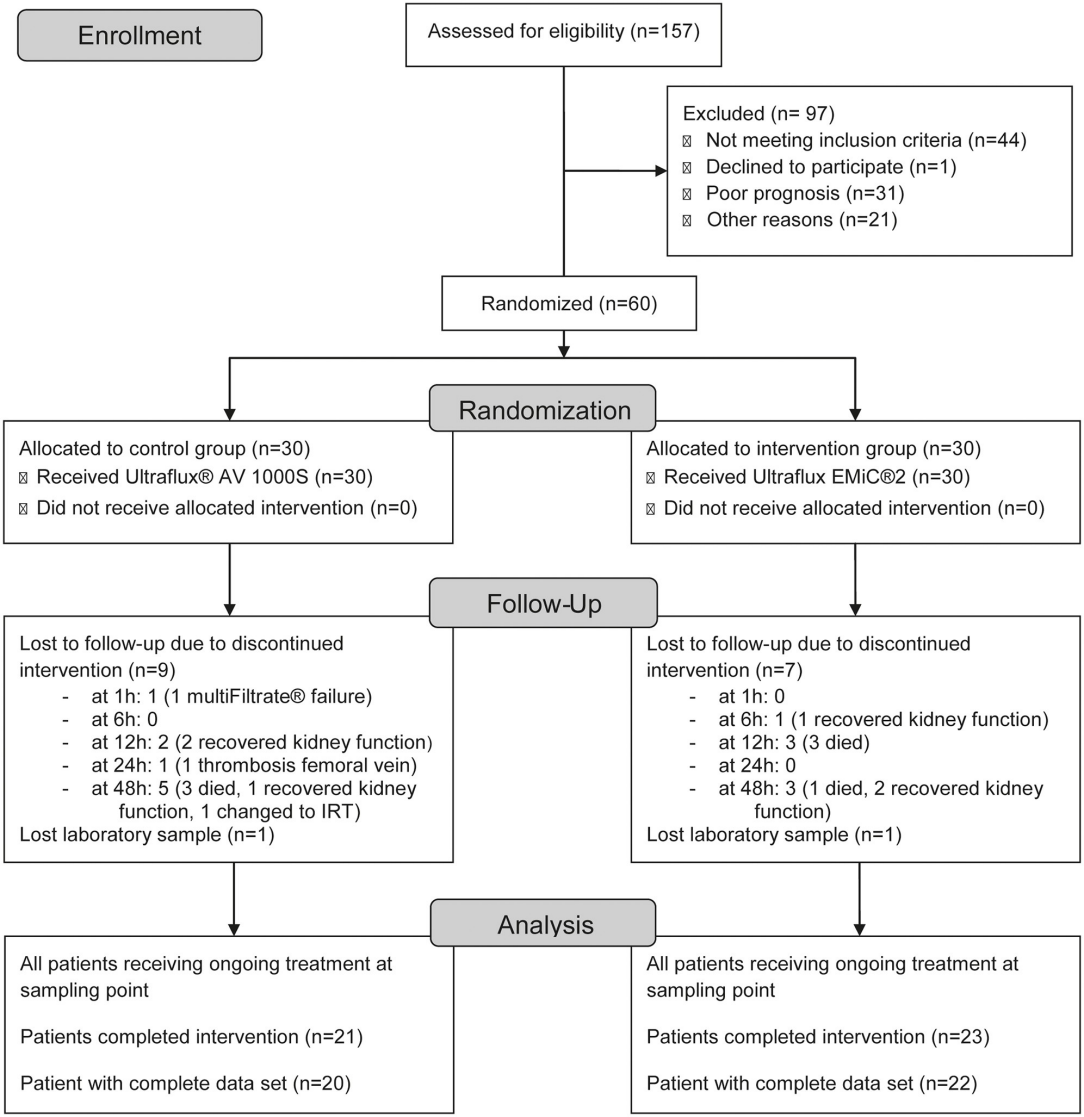
Recruitment flow chart.

Eligible patients were critically ill patients with acute renal failure and indication for RRT based on the recommendations of the Kidney Disease: Improving Global Outcomes [19]. Exclusion criteria were anticoagulation therapy for other reasons, high risk for citrate accumulation (e.g. liver failure), contraindications for renal replacement therapy (e.g. advanced malignant disease), moribund patients, pregnancy and lactation, age lower than 18 years, rejection of renal replacement therapy at all or refusal to participate in the study. 26 patients per group had been calculated to show a difference in plasma clearance of 8 ml/min (two-tailed power 90%, p = 0.05) based on findings by Ricci et al. [22]. Thus, sixty consecutive patients were randomized 1:1 into the intervention and control groups. An unrestricted randomization was carried out using sequentially numbered, opaque sealed envelopes as described by Doig et al. [25]. Recruitment flow chart is illustrated in Fig 1.

The following clinical data were collected in all patients: age, sex, body height, body weight, APACHE II score, SAPS II, SOFA score, the need for mechanical ventilation and vasopressor treatment.

### Intervention

The control group was managed with CVVHD using the high-flux-dialyzer Ultraflux AV 1000S (Fresenius Medical Care, Bad Homburg, Germany), while CVVHD with the HCO-dialyzer Ultraflux EMiC2 (Fresenius Medical Care, Bad Homburg, Germany) was performed in the intervention group. Both dialyzers have the same effective surface area (1.8 m^2^), consist of identical material (polysulfone), exhibit a similar wall thickness (35μm) and differ only in pore size.

Regional citrate anticoagulation was used in both groups. Sodium citrate infusion (citrate: 136 mmol/l) was adjusted based on the concentration of post-dialyzer ionized calcium (target: 0.25–0.34 mmol/l). Calcium chloride solution (calcium: 83 mmol/l) was added to the extracorporeal circuit closer to the vascular access of the patient to keep systemic ionized calcium between 1.12–1.20 mmol/l. The vascular access was in all cases a double-lumen high-flow catheter. RRT was performed with the dialysis machine multiFiltrate (Fresenius Medical Care, Bad Homburg, Germany). A bicarbonate buffered dialysate (Ci-Ca Dialysate K4, Fresenius Medical Care, Bad Homburg, Germany) was used in all cases, at a dose of 25 ml/kg/h after adjusting for body weight. Ideal body weight was calculated using the Hamwi equation (for males: 48 kg for the first 152 cm + 1.1 kg for each additional cm; for females 45 kg for the first 152 cm +0.9 kg for each additional cm; [26]). The quotient of current body weight to ideal body weight was then computed. If this quotient was more than 1.3, the adjusted body weight was used for calculation of dialysate flow (for males: (current body weight-ideal body weight) x 0.38 + ideal body weight; for females: (current body weight-ideal body weight) x 0.32 + ideal body weight; [27]). Blood flow (Q_B_) in the extracorporeal circuit was adjusted threefold of the dialysate flow. Duration of the extracorporeal circuit was limited to 72 hours (h) according to manufacturer`s instructions.

### Endpoints and calculations

The concentrations of urea (60 Dalton (Da)), creatinine (113 Da), β_2_-microglobulin (11800 Da), myoglobin (17053 Da), interleukin 6 (IL-6, 26000 Da) and albumin (66470 Da) were measured pre-and post-dialyzer 1, 6, 12, 24 and 48 h after initiating CVVHD.

Plasma flow in the extracorporeal circuit (Q_p_) was calculated using the patient’s hematocrit level (Hct) at the time of clearance sampling:

$$Q_{p}(\frac{ml}{min})=Q_{B}*\frac{1-Hct}{100}$$

Substance-specific plasma clearance (Cl_p_) at the sampling time points was estimated using the following equation:

$$Cl_{p}(\frac{ml}{min})=Q_{p}*\frac{C_{pre-dialyzer}-C_{post-dialyzer}}{C_{pre-dialyzer}}$$

[28]

The primary outcome was plasma clearance of β_2_-microglobulin, which is considered to represent middle molecules. Secondary endpoints were the plasma clearance of urea, creatinine, myoglobin, IL-6, albumin. For estimating the total clearance (Cl_total_), the integral for substance-specific elimination between the first and the forty-eighth hour was calculated using the following formula:

$$Cl_{total}(\frac{ml}{47h})=\frac{Cl_{1h}+Cl_{6h}}{2}*5*60\;+\;\frac{Cl_{6h}+Cl_{12h}}{2}*6*60\;+\;\frac{Cl_{12h}+Cl_{24h}}{2}*12*60+\;\frac{Cl_{24h}+Cl_{48h}}{2}*24*60$$

The period between starting RRT and the first measurement after 1 h was not considered because of equilibration processes. Mean plasma clearance (Cl_mean_) was determined as follows:

$$Cl_{mean}(\frac{ml}{min})=\frac{Cl_{total}}{47*60}$$

The mean ultrafiltration rate (UFR_mean_) to achieve negative fluid balance was calculated similar mean plasma clearance:

$$UFR_{mean}(\frac{ml}{h})=(\frac{UFR_{1h}+UFR_{6h}}{2}*5+\frac{UFR_{6h}+UFR_{12h}}{2}*6+\frac{UFR_{12h}+UFR_{24h}}{2}*12+\frac{UFR_{24h}+UFR_{48h}}{2}*24)÷47$$

### Laboratory analyses

Blood samples were sent for analysis immediately after the draw. Laboratory analyses were performed using Cobas 8000 (Roche, Mannheim, Germany) according to the manufacturer´s instructions.

The following methods had been used:

- urea: kinetic test with urease and glutamate dehydrogenase
- creatinine: enzymatic method with creatinase
- β_2_-microglobulin: am c701 immunological test for turbidity
- myoglobin: ElektroChemiLumineszenzImmunoAssay (ECLIA)
- IL-6: ECLIA
- albumin: color test with bromocresol green

### Statistical analyses

Data were analyzed using the IBM SPSS 24. Categorical variables were tested by chi-square (two-sided). The Kolmogorov-Smirnov test was performed to test for normal distribution of continuous variables. Normally distributed variables were analyzed by the Student’s *t*-test and presented as mean with standard deviation and confidence interval (CI). Not normally distributed variables were assessed by Mann-Whitney *U* test and presented as median with25^th^ and75^th^ quantiles in brackets. A p value <0.05 was considered statistically significant.

## Results

Baseline characteristics of the study groups are shown in Table 1, with no significant difference between the control group and the intervention group.(Table 1)

**Table 1:** Baseline characteristics.

Variable	control group (n = 30)	intervention group (n = 30)	*p* value
Age, years	59.4±17.7 (52.8–66.1)	63.9±17.4; (57.4–70.4)	0.329
Males	23/30 (76.7%)	22/30 (73.3%)	0.766
Weight, kg	93.5 (78.3,118.5)	80.0 (70.0,95.3)	0.064
Height, cm	174.4±6.7; (171.9–176.9)	172.1±7.3; (169.4–174.8)	0.216
APACHE II	22.1±7.5; (19.3–24.9)	22.4±6.5; (20.0–24.9)	0.841
SOFA-Score	5.4±3.2; (4.2–6.6)	6.4±3.2; (5.2–7.6)	0.214
SAPS II	41.9±16.2; (35.9–48.0)	48.4±15.2; (42.7–54.1)	0.115
Mechanical ventilation	18/30 (60.0%)	19/30 (63.3%)	0.791
Catecholamines	16/30 (53.5%)	19/30 (63.3%)	0.432
Sepsis	13/30 (43.3%)	13/30 (43.3%)	1.000

Data presented as n (%), mean ± standard deviation and 95% confidence interval or median (25^th^, 75^th^ quantile) unless stated otherwise. *Abbreviations*: *CI* 95 percent confidence interval, *APACHE II* Acute Physiology And Chronic Health Evaluation II, *SOFA-score* Sequential Organ Failure Assessment, *SAPS II* Simplified Acute Physiology Score II

The calculated median dialysate flow (control group: 1800 ml/h (1500,2000); intervention group:1800 ml/h (1538,2000) and mean blood flow (control group: 103.6±19.0 ml/min; intervention group: 94.6±18.0 ml/min) did not differ significantly between the treatment groups. Based on this, a dialysate flow of 1800 ml/h (1500,2000) and blood flow of 98 ml/min (90,102.5) in the control group and a dialysate flow of 1900 ml/h (1663,2000) and blood flow of 90 ml/min (80.0,100.0) ml/min in the intervention group were realized without significant differences. The median dialyzer lifespan in the extracorporeal circuit was 69.3h (47.8,73.6) in the control group and 62.8h (49.6,70.1) in the intervention group (p = 0.40). Sodium citrate and calcium chloride infusion rates did not differ between Ultraflux AV 1000S and Ultraflux EMiC2 study arm (Table in S1 Table, Table in S2 Table).

Data for plasma clearance of β_2_-microglobulin are given in Table 2 and Figure in S1 Fig, showing a significant difference between the two groups at all study time points.

**Table 2:** β_2_-microglobulin plasma clearance (ml/min).

Time after starting CVVHD	control group	intervention group	*p* value	n
1h	18.4 (12.0, 23.0)	22.0 (17.3, 30.0)	<0,05	59
6h	13.3±7.7; (CI: 10.4–16.2)	21.9±11.0; (CI: 17.7–26.1)	0.001	58
12h	11.9 (5.2,18.5)	19.0 (13.9, 22.7)	<0,05	53
24h	11.5±6.5; (CI: 8.9–14.1)	17.9±10.2; (CI: 13.8–22.1)	0.009	52
48h	12.1±4.9; (CI: 9.8–14.3)	18.9±11.7; (CI: 13.9–24.0)	0.016	44

Data presented as mean ± standard deviation and 95% confidence interval or median (25th, 75th quantile) unless stated otherwise. *Abbreviations*: *ml/min* milliliters per minute, *CVVHD* continuous veno-venous hemodialysis, *CI* confidence interval

The mean plasma clearance was 12.2±3.6 ml/min (CI: 10.5–13.9) in the control group and 19.6±5.8 ml/min (CI: 17.0–22.1) in the intervention group (p<0.001).

Concerning secondary endpointsa significantly better elimination of myoglobin and IL-6 could be observed in the intervention group, but there was no significant difference for mean urea, creatinine and albumin clearance between the two groups. Mean plasma clearance data are given in Table 3 and Figure in S2 Fig.

**Table 3:** Mean plasma clearance (ml/min), n = 42.

Variable	control group (n = 20)	intervention group (n = 22)	*p* value
Urea	20.7±8.7; (CI: 16.6–24.8)	22.4±7.1; (CI: 19.2–25.5)	0.488
Creatinine	22.9±9.0; (CI: 18.7–27.1)	25.7±8.5; (CI: 21.9–29.5)	0.279
β_2_-microglobulin	12.2±3.6; (CI: 10.5–13.9)	19.6±5.8; (CI: 17.0–22.1)	<0.001
Myoglobin	0.2±3.6; (CI: -1.5–1.9)	8.0±4.5; (CI: 6.0–10.0)	<0.001
IL-6	-2.5±3.5; (CI: -4.1-(-0.9))	1.5±4.3; (CI: -0.4–3.4)	0.002
Albumin	-2.6±4.0; (CI: -4.5-(-0.8))	-2.3±3.9; (CI: -4.1-(-0.6))	0.802

Data presented as mean ± standard deviation and confidence interval. *Abbreviations*: *CI* confidence interval, *ml/min* milliliters per minute, *IL-6* interleukin 6

The median ultrafiltration rate to achieve negative fluid balance showed no significant differences (control group: 50.0 ml/h (0.0, 99.5); intervention group: 12.8 ml/h (0.0, 62.5)). The ratio of β_2_-microglobulin plasma clearance between 1h and 48h to estimate dialyzer performance for middle molecules over time was 1.31 (1.07,1.70) in control group and 1.19 (0.54,1.61) in intervention group without significant differences.

## Discussion

This prospective randomized trial compared the application of two different dialyzers during CVVHD with regional citrate anticoagulation in critically ill patients. A significantly better plasma clearance of middle molecules could be demonstrated with the high cut-off dialyzer compared with the standard high-flux dialyzer. Since both dialyzers are made of the same membrane surface and material, the differences in the plasma clearance of the investigated substances can be explained only by the difference in pore sizes. β_2_-microglobulin is a surrogate parameter for middle molecular uremic toxins. Elevated serum levels of this protein are associated with increased mortality and development of amyloidosis [29, 30, 31, 32]. A few reports showed that reduction in β_2_-microglobulin levels may have mortality benefit in end stage renal disease [29, 30]. Hemodialysis using high cut-off dialyzers was shown to effectively lower plasma β_2_-microglobulin levels [33]. A cross-over study in a small study population using sustained low efficiency daily dialysis (SLEDD) [28] demonstrated a superior elimination of β_2_-microglobulin using the high cut-off Ultraflux EMiC2 dialyzer than with the high-flux dialyzer Ultraflux AV 1000S (plasma clearance: 52 ± 1,7 ml/min vs. 41.7 ± 1.5 ml/min, p<0.001). The higher clearance rate in that study compared to ours is due to the higher volume exchange per time with SLEDD. Similar to that study, our trial also showed that there is no relevant albumin loss with the high cut-off dialyzer. Another investigation showed an enhanced elimination of glutamine and serine with the high cut-off Ultraflux EMiC2 [34], while circulating microRNAs were not eliminated by this dialyzer [35]. A recent study demonstrated a higher clearance of IL-6 and interleukin 10 using Ultraflux EMiC2 compared with AV 1000S in CVVHD with citrate anticoagulation [36]. Another recent trial revealed no differences in removing β_2_-microglobulin between continuous veno-venous hemodiafiltration (CVVHDF) using Ultraflux AV 1000S and CVVHD using Ultraflux EMiC2. However, the dialysis dose was in the CVVHDFgroup (36±4 ml/kg/h) much higher than in the CVVHD group (21±6 ml/kg/h) [37]. In summary, the available data indicate that Ultraflux EMiC2 allows an effective elimination of molecules up to a molecular weight of 40 kDa [38].

Clinical trials with other high cut-off membranes with a maximum cut-off at 50 kDa are limited [39]. An older study on 16 patients with sepsis-related multiple organ failure using an intermittent high permeability hemofiltration over five days for 12 h per day alternated with conventional hemofiltration showed that IL-6 could be eliminated effectively by high cut-off membranes. However, an albumin loss was detected, particularly at the beginning of the RRT, while antithrombin, protein C, protein S, thrombin, coagulation factor V and VIII were not affected [40]. The decline in albumin loss during further RRT could be explained by formation of a new layer at the membrane surface, known as membrane fouling [39]. Our trial showed a significant clearance in IL-6 without albumin loss. This result agrees with the findings of a recent publication [36]. A good dialyzer performance for β_2_-microglobulin over time could be demonstrated in our investigation, which can be explained by a lower membrane fouling and protein cake formation with regional citrate anticoagulation [41].

Several other studies showed that high cut-off dialyzers allow a diffusive removal of cytokines [42, 43, 44]. Therefore, hemodialysis with high cut-off membranes and sufficient dialysate flow seems to be effective in the elimination of inflammatory mediators and safer than high permeability hemofiltration without any relevant albumin loss [39].

A case report on a patient with rhabdomyolysis showed a decline in serum myoglobin levels by 50% within 4 hours using high cut-off intermittent hemodialysis, while myoglobin levels even increased using standard high-flux dialysis [45]. A case series demonstrated that CVVHD and SLEDD with high cut-off dialyzers allow a considerable removal of myoglobin [46]. The present trial found a significantly better elimination of myoglobin in the intervention group. Therefore, patients suffering from rhabdomyolysis and increased risk of bleeding could be treated effectively by citrate anticoagulated CVVHD using the high cut-off Ultraflux EMiC2 dialyzer. Further clinical investigations are required to validate this conclusion.

Other middle molecule toxic proteins may also be removed using the HCO dialyzer. Two case series in patients with multiple myeloma showed a significant removal of free light chains by HCO dialyzer [47, 48]. A case report using the Ultraflux EMiC2 dialyzer showed similar results [38].

There are limitations to our trial. It is a monocentric, single-blinded trial. The study was designed and powered to analyze substance-related elimination in critical care patients. Therefore, the sample size is not adequate to evaluate hard clinical endpoints such as mortality. Furthermore, the dialyzer life span exceeded 48 hours in only 42 of our patients, so that mean plasma clearance calculation was limited to this group. Nevertheless, our study provides a solid background to generate hypotheses and design clinical trials.

## Conclusions

There is an effective elimination of β_2_-microglobulin in citrate anticoagulated CVVHD using the HCO-dialyzer Ultraflux EMiC2 in critical care patients. A significant removal of myoglobin and IL-6 also seems to be possible. Therefore this procedure could be useful in patients suffering from rhabdomyolysis and increased risk of bleeding as well as those with severe inflammation.

## Supporting information

S1 Table Sodium citrate infusion rate. (DOCX)

S2 Table Calcium chloride infusion rate. (DOCX)

S1 Fig β2-microglobulin clearance. (TIF)

S2 Fig Mean plasma clearance. (TIF)

S1 Dataset Data underlying the findings in SPSS format. (SAV)

S2 Dataset Data underlying the findings in EXCEL format. (XLSX)

S1 Protocol Study protocol (english language). (DOC)

S1 Checklist CONSORT Checklist. (DOC)

S1 Supporting Information Abbreviations. (DOCX)

S2 Supporting Information Declarations. (DOCX)
