# Supplementary material for: Middle molecule clearance with high cut-off dialyzer versus high-flux dialyzer using continuous veno-venous hemodialysis with regional citrate anticoagulation: A prospective randomized controlled trial
Source: PLoS One. 2019 Apr 26;14(4):e0215823. doi: 10.1371/journal.pone.0215823 (PMC6485708; doi:10.1371/journal.pone.0215823)
Supplement: S1 Table — (DOCX) [file pone.0215823.s001.docx]

**Sodium citrate infusion rate (mmol sodium citrate/l blood flow)**

| time | control group (n=30) | intervention group (n=30) | *p* value |
| --- | --- | --- | --- |
| 1h | 4.0 (4.0,4.0) | 4.0 (4.0,4.0) | n.s. |
| 6h | 4.0 (4.0,4.0) | 4.0 (4.0,4.0) | n.s. |
| 12h | 4.0 (3.9,4.0) | 4.0 (3.9,4.0) | n.s. |
| 24h | 4.0 (3.8,4.0) | 4.0 (3.9,4.0) | n.s. |
| 48h | 4.0 (3.9,4.2) | 4.0 (3.8,4.0) | n.s. |

Data presented as median (25^th^, 75^th^ quantile). *Abbreviations:* *n.s.* not significant
