# Supplementary material for: Middle molecule clearance with high cut-off dialyzer versus high-flux dialyzer using continuous veno-venous hemodialysis with regional citrate anticoagulation: A prospective randomized controlled trial
Source: PLoS One. 2019 Apr 26;14(4):e0215823. doi: 10.1371/journal.pone.0215823 (PMC6485708; doi:10.1371/journal.pone.0215823)
Supplement: S1 Protocol — (DOC) [file pone.0215823.s007.doc]

Middle molecule clearance with high cut-off dialyzer versus high-flux dialyzer using continuous veno-venous hemodialysis with regional citrate anticoagulation (CIMIC)

Principal investigator:

Lorenz Weidhase, M.D.

University Hospital Leipzig

Medical ICU

Director: Sirak Petros, M.D., Ph.D.

Liebigstraße 20

Leipzig, Germany

Tel.: ++49 341 9712706

Fax.: ++49 341 9712709

email: [Lorenz.Weidhase@medizin.uni-leipzig.de](mailto:Lorenz.Weidhase@medizin.uni-leipzig.de)

Study sub-investigators:

Gero Schulze, M.D.

Alexander Peschka, M.D.

Further members of the study group:

Ludger Mende, M.D.

Katrin Knoll, M.D.

Frank Seidel, M.D.

Elena Dafova, M.D.

Martin Grimm, M.D.

Jonathan De Fallois, M.D.

Christine Göttel

Beatrice Vogt

**Abbreviations used in the protocol**

APACHE II Score: Acute Physiology And Chronic Health Evaluation II Score

BGA: Blood gas analysis

C: concentration

CE: administrative marking for medical products approved in the European Union

Cl: Clearance

Cl_total_: total clearance between the first hour and the end of 48 hours

Cl_mean_: mean plasma clearance

CRP: C-reactive protein

CVVH: continuous veno-venous hemofiltration

CVVHD: continuous veno-venous hemodialysis

CRRT: continuous renal replacement therapy

EMiC: Enhanced Middle Molecule Clearance

Hb: Hemoglobin

Hct: Hematocrit

IHD: intermittent hemodialysis

IL-6: Interleukin-6

KDIGO: Kidney Disease: Improving Global Outcomes

PCT: Procalcitonin

Q_B_: Blood flow

Q_P_: Plasma flow

RRT: renal replacement therapy

SAPS II-Score: Simplified Acute Physiology Score

SOFA-Score: Sequential Organ Failure Assessment Score

SPSS: Superior Performing Software System

**1. Introduction**

In critically ill patients, acute renal failure with a need for renal replacement therapy (RRT) is associated with a mortality rate of more than 50% (1). Acute renal failure is an independent risk factor for death in this patient population (2). Continuous renal replacement therapy (CRRT) is considered to have the theoretical advantage of better hemodynamic tolerance and effective fluid removal (3). However, it is not associated with a survival benefit compared to intermittent hemodialysis (IHD) (4). The current evidence does not allow any clear recommendation regarding the optimal dose (5, 6, 7, 8) and the best time point for starting RRT (3).

During CRRT, systemic anticoagulation to maintain the extracorporeal circuit, particularly in patients with an increased bleeding risk, can be a considerable challenge. In the last decade, regional citrate anticoagulation has been introduced as a practical and safe means for selective anticoagulation of the extracorporeal circuit (9). Citrate hemodialysis is associated with less bleeding (10), longer filter life span (11) and possibly improved survival (12). Therefore, citrate hemodialysis is generally recommended in the current KDIGO guidelines if systemic anticoagulation is not required for other reasons and there is no contraindication against citrate application.

Citrate is metabolized to bicarbonate in the intermediate metabolism. However, the physiologic citrate metabolism may be impaired in patients with reduced liver function, such as in septic shock or decompensated cirrhosis of the liver. Significantly lower blood flow, therefore less citrate, is required in hemodialysis than in hemofiltration. Therefore, regional citrate anticoagulation has been limited to continuous veno-venous hemodialysis (CVVHD) in order to reduce the citrate load to the critically ill. However, compared to continuous veno-venous hemofiltration (CVVH), middle molecule clearance is inadequate with CVVHD (13), which may be unfavorable in septic patients. A special high-cutoff dialyzer (EMiC®2) has thus been developed to overcome this issue.

The aim of this prospective randomized single-center trial is to investigate the middle molecule clearance rates with CVVHD using high-cutoff EMiC®2 dialyzer versus those of CVVHD using the classic high-flux dialyzer, both with regional citrate anticoagulation. The concept of citrate-based CVVHD with the EMiC®2 dialyzer may be an option to combine the advantages of the classic citrate CVVHD and CVVH. The trial is investigator-initiated and it will be conducted according to the German medical device law.

**2. Endpoints**

**Primary endpoint**: Does a citrate-based CVVHD with the EMiC®2 high-cutoff dialyzer lead to a better middle molecule clearance than a citrate-based CVVHD with a high-flux dialyzer, with β_2_-microglobulin as a marker molecule?

**Secondary endpoint:** Clearance rates of other molecules with different molar masses (urea, creatinine, myoglobin, IL-6 and albumin) using the EMiC®2 high-cutoff dialyzer compared to that of CVVHD with high-flux dialyzer.

**3. Patients and Methods**

Critically ill patients fulfilling the criteria for RRT will be consecutively screened (see inclusion criteria below). Using a 1:1 envelope randomization, patients will be assigned to either classic high-flux citrate-based CVVHD (group 1) or to citrate-based CVVHD using the high-cutoff EMiC®2 dialyzer (group 2). Patients who are screened but excluded from the trial will be recorded in a log book.

After inserting a hemodialysis catheter, CVVHD (multi**Filtrate®, Fresenius, Germany)** will be started using either the **polysulfone** Ultraflux® AV 1000S **high-flux dialyzer in group 1 or the polysulfone** Ultraflux EMiC®2 high-cutoff dialyzer in group 2 (both from Fresenius Medical care, Germany)**. A bicarbonate-buffered dialysate (Ci-Ca® Dialysate K4, Fresenius Medical Care, Germany**) will be used.

Regional anticoagulation in the extracorporeal circuit will be achieved through modification of the post-filter ionized calcium (Ca^2+^) concentration by adding a citrate solution (target post-filter Ca^2+^: 0.25 – 0.3 mmol/l).

The dialysate flow will be 25 ml/kg body weight (14). The blood flow (Q_B_) will be adjusted at threefold of the dialysate flow. A calculator program will be available for the study group. The extent of the ultrafiltration will be adjusted based on the condition of the patient and the decision of the treating physician. The dialysis system will be changed every 72 hours at the latest according to the instructions of the manufacturer.

Simultaneous measurements of urea, creatinine, β_2_-microglobulin, myoglobin, interleukin-6 and albumin will be conducted 1 (t0), 6 (t1), 12 (t2), 24 (t3) and 48 hours (t4) after the start of CVVHD from samples drawn immediately before (pre-filter) and after the dialyzer (post-filter). The samples will be provided with a study-specific code and immediately sent to the laboratory.

The plasma flow (Q_p_) will be calculated using the following formula based on the individual hematocrit:

Q_p_= Q_B_ x (1-Hct/100)

The substance-specific clearance (Cl) due to RRT at the above mentioned time points will then be calculated using the following formula:

Cl (ml/min) = Q_P_ x ([C_prefilter_- C_postfilter_]/ C_prefilter_)

The total clearance (Cl_total_) from t0 to t4 will then be computed using the following formula:

Cl_total_ = (((Cl_1h_ + Cl_6h_) / 2) * 5 * 60) + (((Cl_6h_+ Cl_12h_) / 2) * 6 * 60) + (((Cl_12h_+ Cl_24h_) / 2) *

12 * 60) + (((Cl_24h_ + Cl_48h_) / 2) * 24 * 60)

The mean plasma clearance (Cl_mean_) in ml/min will then be derived from the above data as follows:

Cl_mean_ = Cl_total_ /47/60

Furthermore, data for filter life span, duration of RRT, interruptions of RRT as well as the course of markers of systemic inflammation will be collected.

Based on statistical calculations from pilot data, a sample size of 60 with 30 patients in each arm is planned.

**Inclusion criteria**

The inclusion criteria are based on the current KDIGO guidelines for initiation of extracorporeal renal support. RRT should be started as soon as possible in patients with life-threatening water, electrolyte and/or acid-base disorders (14)

**Exclusion criteria**

- Therapeutic anticoagulation required for reasons other than RRT
- Age <18 years
- Expected high risk for citrate accumulation (e.g. severe acute liver failure)
- RRT considered contraindicated (e.g. terminal malignant disease) or rejected by the patient
- Morbid patient with a high likelihood to die within 48 hours
- Pregnancy and lactation
- Participation in another trial
- Refusal to participate in the study

RRT will not be delayed for the sake of this trial. If a patient cannot be included in the trial at the beginning of the first RRT course, a study inclusion is still possible at a later time, for example after the regular filter change, if the indication for RRT persists.

**Inclusion algorithm**

Indication for CRRT

Patient can give consent

Patient is not able to give consent

Informed consent given

No consent

Study inclusion

No study inclusion

Legal guardian is available

No legal guardian

Urgent institution of a legal guardian

**Risk-benefit assessment**

Improved clearance of larger molecules with CVVHD using the high-cutoff dialyzer EMiC®2, combining the benefits of regional citrate anticoagulation and CVVH, may possibly have an advantage in certain disease states, such as sepsis or rhabdomyolysis. Based on current knowledge, specific risks due to this intervention are not expected. The EMiC®2 dialyzer is approved for citrate-based hemodialysis and it has already received the official CE marking.

A maximum of 50 ml blood sample is required within the maximum study CRRT period of 48 hours. These blood samples will be drawn from the extracorporeal circuit, thus pain-free to the patient. Further study procedures are not planned.

**4. Data compilation**

Patient data will be documented anonymized in a data base. Additional to the clearance variables mentioned earlier, the following data will be documented for every patient:

- Age, gender, height, weight
- Major diagnosis
- Indication for RRT
- ICU scores
  - on study inclusion: APACHE II score, SAPS II score, SOFA score
  - daily: SOFA score, SAPS II score until ICU discharge
- pre-existent end-stage renal disease (predefined subgroup analysis)
- location of the vascular access
- daily laboratory assessment (at 5 am):
  - hemoglobin, hematocrit, white cell count, platelet count
  - blood urea, creatinine
  - Na^+^, K^+^, Cl^-^, PO_4_^3-^, Ca^2+^, Mg^2+^
  - BGA every 6 hours
  - Serum CRP, PCT, IL-6, albumin
- Hemodialysis protocol with 6-hourly documentation of blood flow (ml/min), dialysate flow (ml/h), ultrafiltration (ml/h), citrate infusion rate, calcium substitution rate, transmembrane pressure, pre- and post-filter Ca^2+^.
- Filter life span
- Duration of RRT interruptions
- ICU and hospital length of stay
- ICU, hospital, 28- and 90-day survival
- Status of renal function (>96 hours RRT-free period)

The statistical analysis will be carried out using the program package SPSS for Windows.

**Funding**

This trial is funded by an unrestricted grant from Fresenius Medical Care Deutschland GmbH, Else-Kröner-Straße 1, D-61352 Bad Homburg, Germany. The sponsor does not have any influence on any part of the trial.

**Signature**

Lorenz Weidhase, MD Sirak Petros, MD, PhD

Principal investigator Director, Medical ICU

**5. Literature**

1. [Uchino S](http://www.ncbi.nlm.nih.gov/pubmed?term="Uchino S"%5BAuthor%5D), [Kellum JA](http://www.ncbi.nlm.nih.gov/pubmed?term="Kellum JA"%5BAuthor%5D), [Bellomo R](http://www.ncbi.nlm.nih.gov/pubmed?term="Bellomo R"%5BAuthor%5D), et al. Beginning and Ending Supportive Therapy for the Kidney [(BEST Kidney) Investigators](http://www.ncbi.nlm.nih.gov/pubmed?term="Beginning and Ending Supportive Therapy for the Kidney (BEST Kidney) Investigators"%5BCorporate Author%5D). Acute renal failure in critically ill patients: a multinational, multicenter study. [JAMA.](javascript:AL_get(this, 'jour', 'JAMA.');) 2005 Aug 17;294(7):813-8.
2. Levy EM, Viscoli CM, Horwitz RI. The effect of acute renal failure on mortality. A cohort analysis. JAMA. 1996 May 15;275(19):1489-94.
3. Palevsky PM. [Dialysis modality and dosing strategy in acute renal failure.](http://www.ncbi.nlm.nih.gov/pubmed/16551296) Semin Dial. 2006 Mar-Apr;19(2):165-70.
4. Pannu N, Klarenbach S, Wiebe N, Manns B, Tonelli M; Alberta Kidney Disease Network. Renal replacement therapy in patients with acute renal failure: a systematic review. JAMA. 2008 Feb 20;299(7):793-805.
5. Ronco C, Bellomo R, Homel P, Brendolan A, Dan M, Piccinni P, La Greca G. Effects of different doses in continuous veno-venous haemofiltration on outcomes of acute renal failure: a prospective randomised trial. Lancet. 2000 Jul 1;356(9223):26-30.
6. Saudan P, Niederberger M, De Seigneux S, et al. Adding a dialysis dose to continuous hemofiltration increases survival in patients with acute renal failure. Kidney Int. 2006 Oct;70(7):1312-7.
7. VA/NIH Acute Renal Failure Trial Network, Palevsky PM, Zhang JH, O'Connor TZ, et al. [Intensity of renal support in critically ill patients with acute kidney injury.](http://www.ncbi.nlm.nih.gov/pubmed/18492867) N Engl J Med. 2008 Jul 3;359(1):7-20. Erratum in: N Engl J Med. 2009 Dec 10;361(24):2391.
8. RENAL Replacement Therapy Study Investigators, Bellomo R, [Cass A](http://www.ncbi.nlm.nih.gov/pubmed?term=Cass A%5BAuthor%5D&cauthor=true&cauthor_uid=19846848), [Cole L](http://www.ncbi.nlm.nih.gov/pubmed?term=Cole L%5BAuthor%5D&cauthor=true&cauthor_uid=19846848), et al. Intensity of continuous renal-replacement therapy in critically ill patients. N Engl J Med. 2009 Oct 22;361(17):1627-38.
9. M[orgera S](http://www.ncbi.nlm.nih.gov/pubmed?term=Morgera S%5BAuthor%5D&cauthor=true&cauthor_uid=16155399), Haase M, [Ruckert M](http://www.ncbi.nlm.nih.gov/pubmed?term=Ruckert M%5BAuthor%5D&cauthor=true&cauthor_uid=16155399), et al. Regional citrate anticoagulation in continuous hemodialysis--acid-base and electrolyte balance at an increased dose of dialysis. [Nephron Clin Pract.](http://www.ncbi.nlm.nih.gov/pubmed/16155399#%23) 2005;101(4):c211-9.
10. [Gabutti L](http://www.ncbi.nlm.nih.gov/pubmed?term=Gabutti L%5BAuthor%5D&cauthor=true&cauthor_uid=12373466), [Marone C](http://www.ncbi.nlm.nih.gov/pubmed?term=Marone C%5BAuthor%5D&cauthor=true&cauthor_uid=12373466), [Colucci G](http://www.ncbi.nlm.nih.gov/pubmed?term=Colucci G%5BAuthor%5D&cauthor=true&cauthor_uid=12373466), [Duchini F](http://www.ncbi.nlm.nih.gov/pubmed?term=Duchini F%5BAuthor%5D&cauthor=true&cauthor_uid=12373466), Schönholzer C.: Citrate anticoagulation in continuous venovenous hemodiafiltration: a metabolic challenge. [Intensive Care Med](http://www.ncbi.nlm.nih.gov/pubmed?term=Gabutti et al.%2C Intensive Care Med 28%3A1419–1425%2C 2002" \l "%23). 2002 Oct;28(10):1419-25.
11. [Morgera S](http://www.ncbi.nlm.nih.gov/pubmed?term=Morgera S%5BAuthor%5D&cauthor=true&cauthor_uid=15331942), [Scholle C](http://www.ncbi.nlm.nih.gov/pubmed?term=Scholle C%5BAuthor%5D&cauthor=true&cauthor_uid=15331942), [Voss G](http://www.ncbi.nlm.nih.gov/pubmed?term=Voss G%5BAuthor%5D&cauthor=true&cauthor_uid=15331942), et al. Metabolic complications during regional citrate anticoagulation in continuous venovenous hemodialysis: single-center experience. [Nephron Clin Pract](http://www.ncbi.nlm.nih.gov/pubmed?term=Morgera et al.%2C Nephron Clin Pract 97%3Ac131-c136%2C 2004" \l "%23). 2004;97(4):c131-6.
12. Oudemans-van Straaten HM, Bosman RJ, Koopmans M, et al. [Citrate anticoagulation for continuous venovenous hemofiltration.](http://www.ncbi.nlm.nih.gov/pubmed/19114912) Crit Care Med. 2009 Feb;37(2):545-52.
13. Ricci Z, Ronco C, Bachetoni A, et al. Solute removal during continuous renal replacement therapy in critically ill patients: convection versus diffusion. Crit Care. 2006;10(2):R67.
14. Kidney Disease: Improving Global Outcomes (Kdigo) Acute Kidney Injury Work Group (2012) KDIGO clinical practice guideline for acute kidney injury. Kidney Int 2(1):1–138.
