# Supplementary material for: Middle molecule clearance with high cut-off dialyzer versus high-flux dialyzer using continuous veno-venous hemodialysis with regional citrate anticoagulation: A prospective randomized controlled trial
Source: PLoS One. 2019 Apr 26;14(4):e0215823. doi: 10.1371/journal.pone.0215823 (PMC6485708; doi:10.1371/journal.pone.0215823)
Supplement: S1 Supporting Information — (DOCX) [file pone.0215823.s009.docx]

**Abbreviations**

RRT: renal replacement therapy; CRRT: continuous renal replacement therapy; IHD: intermittent hemodialysis; CVVHD: continuous veno-venous hemodialysis; CVVH: continuous veno-venous hemofiltration; HCO: high cut-off; ICU: intensive care unit; KDIGO: Kidney Disease: Improving Global Outcomes; Q_B_: blood flow in extracorporeal circuit; h: hour; Da: Dalton; kDa: kilo-Dalton; Q_p_: plasma flow in extracorporeal circuit; Hct: hematocrit; Cl_p_: plasma clearance; ml/min: milliliters per minute; ml/h: milliliters per hour; C_post-dialyzer_: concentration post-dialyzer; C_post-dialyzer, corr._: corrected concentration post-dialyzer; C_pre-dialyzer_: concentration pre-dialyzer; Cl_total_: total plasma clearance; Cl_1h_: plasma clearance after 1 hour; Cl_6h_: plasma clearance after 6 hours; Cl_12h_: plasma clearance after 12 hours; Cl_24h_: plasma clearance after 24 hours; Cl_48h_: plasma clearance after 48 hours; Cl_mean_: mean plasma clearance; UFR_mean_: mean ultrafiltration rate; UFR_1h_: ultrafiltration rate after 1 hour; UFR_6h_: ultrafiltration rate after 6 hours; UFR_12h_: ultrafiltration rate after 12 hours; UFR_24h_: ultrafiltration rate after 24 hours; UFR_48h_: ultrafiltration rate after 48 hours; CI: confidence interval; APACHE II: Acute Physiology And Chronic Health Evaluation II ; SAPS II: Simplified Acute Physiology Score II; SOFA score: Sequential organ failure assessment score; RNA: ribonucleic acid; SLEDD: sustained low-efficiency daily dialysis; CVVHDF: continuous veno-venous hemodiafiltration
