# Supplementary material for: Middle molecule clearance with high cut-off dialyzer versus high-flux dialyzer using continuous veno-venous hemodialysis with regional citrate anticoagulation: A prospective randomized controlled trial
Source: PLoS One. 2019 Apr 26;14(4):e0215823. doi: 10.1371/journal.pone.0215823 (PMC6485708; doi:10.1371/journal.pone.0215823)
Supplement: S2 Supporting Information — (DOCX) [file pone.0215823.s010.docx]

**Declarations**

**Ethics approval and consent to participate**

The study was approved by the local ethics committee (University of Leipzig, reference number: 447-12-24092012), conducted in accordance with the German medical product law and registered in the German Clinical Trials Register (DRKS00005254, registered 26th November 2013). Informed consent was obtained from all participating subjects.

**Consent for publication**

For all participants we obtained informed consent by the patients themselves or their legal guardians.

**Availability of data and materials**

The datasets used and/or analyzed during the current study are available from the corresponding author on request.

**Competing interests**

The authors declare that they have no competing interests.

**Funding**

Laboratory analyses, medical disposables and additional costs were funded by Fresenius Medical Care Deutschland GmbH, Else-Kröner-Straße 1, D-61352 Bad Homburg v.d.H. This is an investigator initiated trial. The funders had no role in study design, data collection and analysis, decision to publish, or preparation of the manuscript. There is no relation of authors to the funder concerning employment, consultancy, patents, products in development and marketed products. This does not alter our adherence to PLOS ONE policies on sharing data and materials.

**Authors’ contributions**

LW: conception, design, literature research, statistical analysis, writing the manuscript

EH: acquisition of date, documentation of data, literature research, statistical analysis

SH: revision of the manuscript, statistical analysis

TK: laboratory analysis

JF: acquisition of date, revision of the manuscript

SP: revision of the manuscript

All authors gave final approval of the final manuscript to be published.

**Authors’ information**

Not applicable.
